# Host Responses in Peri-implant Tissue in Comparison to Periodontal Tissue: A Retrospective Study

**DOI:** 10.3290/j.ohpd.b2585655

**Published:** 2022-01-20

**Authors:** Pravej Serichetapongse, Raviporn Madarasmi, Anjalee Vacharaksa

**Affiliations:** a Associate Professor, Department of Prosthodontic, Esthetic, Restorative and Implant Dentistry Clinic, Faculty of Dentistry, Chulalongkorn University, Bangkok, Thailand. Project planning, proposal preparation, manuscript preparation.; b Graduate Student, Esthetic, Restorative and Implant Dentistry Program, Faculty of Dentistry, Chulalongkorn University, Bangkok, Thailand. Proposal preparation, data collection, statistical analysis, manuscript preparation.; c Assistant Professor, Department of Microbiology and Research Unit on Oral Microbiology and Immunology, Geriatric Dentistry and Special Patients Care Clinic, Faculty of Dentistry, Chulalongkorn University, Bangkok, Thailand. Proposal preparation, data collection, statistical analysis, manuscript preparation.

**Keywords:** cytokines, fibroblast, gold alloy, inflammatory response, peri-implant fluid, titanium

## Abstract

**Purpose:**

To investigate inflammatory responses in peri-implant crevicular fluid (PICF) in comparison to periodontal tissue.

**Materials and Methods:**

Nineteen participants with healthy implants restored with titanium or gold-casting abutment were included. PICF and gingival crevicular fluid (GCF) were collected for inflammatory cytokine detection by ELISA. Cytokine levels in PICF or GCF of the same individual were compared using the paired t-test, and those from titanium or gold-casting (UCLA) abutment were compared using the independent t-test. Human gingival fibroblast responses to PICF and GCF were then evaluated with one-way ANOVA.

**Results:**

The results demonstrated that IL-6, IL-8, TNFα, and IFNγ expressed in PICF are similar to GCF in the same individual. However, IL-1β (p = 0.032) and IL-1α (p = 0.030) was statistically significantly higher in PICF than in GCF. IL-8 level was statistically significantly higher with gold-casting than with titanium abutments (p = 0.003). PICF statistically significantly stimulated higher expression of RANKL, IL-1β, IL-6, and IL-8 mRNA in human gingival fibroblasts (HGF), while focal adhesion kinase (FAK) suppressed mRNA.

**Conclusion:**

The inflammatory cytokines, including IL-1α and IL-1β, are higher in healthy peri-implant tissues. Abutment materials may also influence the level of inflammatory cytokines in PICF. Inflammatory mediators in crevicular fluid may affect HGF inflammatory responses and peri-implant tissue integration.

Implant-supported prostheses have become the treatment of choice to restore function and aesthetics in partially or completely edentulous arches, thanks to high success rates and excellent long-term value.^4,24^ Although dental implants share some similarities with natural teeth, they also differ in many ways, and that makes peri-implant tissue respond differently to microbial challenges.^9^ Upon exposure to the oral environment, implant surfaces are immediately colonised by oral bacteria.^8^ After successful tissue healing, mucosal epithelium represents the first line of defense against external stimuli. The soft tissue surrounding the implant surface forms a deeper sulcus than that found around healthy natural teeth.^3^ Oral biofilm therefore forms in close physical contact to the bone-implant interface, and this increases the chance of host/microbial interactions. The connective tissue attachment of dental implants differs from that of natural teeth: collagen fibers extend from the bone and lack attachment to the implant surface.^3^ Thus, connective tissue surrounding dental implants has less mechanical resistance compared with natural teeth. The possibility of attachment breakdown leading to tissue loss is greater with dental implants when faced with a bacterial challenge. The dental implant and its restoration surfaces selectively support initial bacterial adhesion and determine biofilm formation; implant- and tooth-associated biofilms differ.^15^ In addition to peri-implant microbiota, the micro-environment around dental implants may be influenced by restorative and dental implant materials. Differences in surface and material type have a stronger influence on cellular adhesion and biofilm formation on dental implants compared to natural teeth. For example, the titanium oxide layer may be dissolved in the oral environment through a corrosion cycle,^7^ while titanium-particle– or ion-induced foreign body reactions were reported in an animal model.^29^ Conversely, it is important for host responses in peri-implant tissue to strengthen the epithelial integrity of dental implants to maintain tissue homeostasis when dental implants are in function.

The transfer of biological knowledge about peri-implantitis and periodontitis should help to develop new biological understanding about both health and disease of teeth and dental implants. In this study, we investigated the host response in peri-implant tissue in comparison to the host response in periodontal tissue. Moreover, the differences in host response between two abutment materials were also reported. This may provide baseline understanding of host responses in peri-implant tissue and the responses to differential implant abutments.

## Materials and Methods

### Participant Selection

The study was approved by the Ethics Subcommittee, Faculty of Dentistry, Chulalongkorn University, Bangkok, Thailand (HREC-DCU 2016-046). Participants who received implant-supported fixed partial prostheses from the Esthetic, Restorative, and Implant Dentistry Clinic, Chulalongkorn University, from January 2010 to December 2016 were included in the study. Participants were divided into groups according to abutment materials used: the UCLA (gold/palladium alloy; Basel, Switzerland) group and the titanium abutment group. Following the inclusion and exclusion criteria, individuals with a healthy single implant placed in the anterior sextant with controlled oral hygiene and with positive informed consent were enrolled. Individuals with a history of smoking, myocardial infarction within 6 months, history of radiotherapy in the head and neck region, antitumor chemotherapy, liver diseases, blood diseases, kidney diseases, immunosuppressed status, current corticosteroid therapy, on antibiotics within the past 3 months, pregnancy, inflammatory and autoimmune diseases of the oral cavity were excluded.

Periodontal parameters such as bleeding on probing (BOP), gingival index (GI), plaque index (PI) and probing depth (PD) were evaluated (Periowise, Premier Dental; Plymouth Meeting, PA, USA). The Silness and Löe plaque and gingival inflammation indices were used to measure the amount of plaque and gingival inflammation present on four surfaces of a tooth. Intraoral radiographs were taken on implants using the periapical technique to ensure no bone loss occurred up to the first thread of the implant.

### Peri-implant Fluid and Gingival Crevicular Fluid Collection

For peri-implant (PICF) and gingival crevicular fluid (GCF) collection, the tooth with the same probing depth as the peri-implant was chosen for the collection of GCF. The site for peri-implant and gingival crevicular fluid collection was isolated using a cotton roll and air dried to ensure good moisture control. Supragingival calculus or plaques were removed 2 weeks prior to sample collection. PICF and GCF were collected using four sterile absorbent paper points size M (Kerr; Orange, CA, USA). The absorbent paper point were marked at 15 mm and then placed in the sulcus for 30 s. PICF absorbed from each strip was stored in 1.5-ml plastic tubes containing 100 μl of phosphate-buffered saline (PBS), pH 7.2, supplemented with protease inhibitor cocktail (Roche Diagnostics; Mannheim, Germany). These were then centrifuged at 8000 rpm for three min, after which the paper points were discarded, and the supernatants were stored at -80°C until use.

### Single-Analyte ELISArray Kits

The single-analyte ELISArray kit was designed to quantitatively measure the amount of IL-1α, IL-1β, IL-6, IL-8, IFN-γ and TNF-α using a standard sandwich enzyme-linked immunosorbent assay (ELISA) technique. A target-specific capture antibody was coated on the plate. The single-analyte ELISArray kit contains assay buffer, detection antibody, avidin-HRP and a 96-well plate. ELISA was performed according to the manufacturer’s instructions (Qiagen; Valencia, CA, USA).

### Human Gingival Fibroblast (HGF) Cell Culture

After informed consent, gingival tissue for primary gingival fibroblast culture was obtained from healthy human subjects (age 14 to 40 years) who needed crown-lengthening surgery at the Esthetic, Restorative, and Implant Dentistry Clinic (HREC-DCU 2016-046). The tissue specimens were washed 3 times with PBS containing antibiotics, before being cut into small pieces and placed on the culture plate (Corning; New York, NY, USA) containing DMEM supplemented with 10% heat-inactivated foetal bovine serum (FBS), 100 U/ml penicillin, 100 μg/ml streptomycin and 1% amphotericin B (Thermo Fisher Scientific; Waltham, MA, USA) to prevent growth of microorganisms. The primary cultures were incubated at 37°C in a humidified atmosphere of 5% CO_2_. The medium was changed every two days. When the primary cell culture reached a confluence of 70%-80%, the HGF were detached with 0.025% trypsin-EDTA (Thermo Fisher Scientific), and 0.05% trypsin-EDTA, diluted with culture medium and then subcultured at a ratio of 1:3. HGF from passages three to seven were used in the experiment. 3x10^4^ cells were seeded in 6-well plates (Corning) and cultured for 24 h prior to the treatment. PICF from dental implants restored with titanium or UCLA abutment and GCF from each individual were pooled together at equal volume%. HGF cultures were incubated with 0.005%, 0.01% and 0.05% volume of pooled PICF restored with titanium or pooled UCLA abutment, or pooled GCF, respectively. After the treatment, cells were incubated at 37°C in a humidified atmosphere of 5% CO_2_ for 24 h. Some wells were incubated with *P. gingivalis* LPS (0.1 μg/ml) for 24 h for positive control. Cells were then harvested for RNA extraction, and when necessary, stored at -80°C. Each experiment was performed in triplicate wells, and three independent experiments were repeated with HGF from three different individuals.

### RNA Isolation and Quantitative RT-PCR

Total RNA was extracted with TRIzol reagent (Molecular Research Center; Cincinnati, OH, USA) according to manufacturer’s instructions. Briefly, 1 ml of TRIzol reagent was added to each culture well. Then, TRIzol reagents were transferred into 1.5-ml tubes, 200 µl of chloroform were added, then the tubes were shaken vigorously. The mixtures were then centrifuged at 14,000 rpm for 15 min, after which the aqueous phase was collected. Isopropanol (500 µl) was added to precipitate RNA. After centrifugation, pellets were dissolved in nuclease-free water, and the amount of RNA was determined by the absorption at 260/280 nm using a micro-Volume UV-Vis Spectrophotometer for Nucleic Acid and Protein Quantitation (NanoDrop2000, Thermo Fisher Scientific).

One µg of RNA was converted to cDNA by Improm-IITM reverse transcriptase system (Promega; Madison, WI, USA) as recommended by the manufacturer. Subsequently, quantitative PCR (qPCR) was performed by using QuantiTect SYBR Green PCR Kits (Qiagen). Primers sequences used in this study are shown in Table 3. PCR amplification of the cDNA template was performed using LightCycler 480 SYBR Green I Master kit (Roche Diagnostics) on MiniOpticon Real-Time PCR Detection System (Bio-Rad; Hercules, CA, USA). PCR conditions were 95°C for 1 min followed by 40 cycles of amplification at 95°C for 10 s, 60°C for 10 s, and 72°C for 20 min. The reactions were performed in duplicate, and the average values were used for gene expression analysis. The data for comparative analysis of gene expression was obtained using the Ct method. Glyceraldehyde-3-phosphate dehydrogenase (GAPDH) mRNA expression was used as an internal control. The qPCR products were stained with ethidium bromide on a 1.8% agarose gel to confirm the specific product size.

### Statistical Analysis

The data were analysed by using the SPSS program (SPSS version 16.0; Chicago, IL, USA). A normal distribution of all data was tested. The paired sample t-test was used to compared cytokine concentrations between PICF (implants) and GCF (natural teeth). The independent t-test was used to compare the concentration of cytokines between UCLA and titanium abutments. One-way ANOVA was used to determine statistically significant differences in FAK, RANKL, IL-1β, IL-8 and TNFα gene expression between fibroblast cells treated with PICF obtained around titanium and UCLA abutments.

## Results

All participants had healthy dental implants restored with single-unit prostheses. Ten participants had a restoration with a UCLA abutment, while 9 had a titanium abutment. A pristine tooth without any restoration located in the same sextant of the implant was selected for the GCF sample collection. The demographic data and clinical parameters of implants and teeth are shown in Tables 1 and 2, respectively. The mean age (± SD) of all participants was 47.5 (± 11.12). The implants and neighbouring teeth appeared in a healthy state, showing no bleeding on probing. Progressive bone resorption was not observed in any participant. The average (± SD) loading time of implants in function was 49.7 (± 19.1) months. Specifically, the average time in function of implants restored with titanium abutments (n = 9) was 60.2 (± 9.8) months, and that of implants restored with UCLA abutments (n = 10) was 40.2 (± 22.1) months.

**Table 1:** Demographic data of participants and characteristics of implant-supported prostheses

Participants (n)	19
Age (years)	42.84 (± 13.07), 45.5^#^
**Gender**	
Male (n)	9
Female (n)	10
Implant-supported crown (n)	19
**Implant system**	
Astra Tech-Titanium(n)	8
Straumann-Titanium (n) Straumann-UCLA (n)	1 10
Loading time (months) Titanium abutment UCLA abutment	49.7 (+19.1) (20–67)^#^ 60.2 (+9.8) 40.2 (+22.1)
Distance between implant shoulder-to-bone contact (mm)	2.78 (+0.78), 2.62^#^

^#^Mean (± SD), median.

**Table 2:** Clinical parameters (mean ± SD)

Score	Titanium abutment		UCLA abutment	
	Tooth	Implant	Tooth	Implant
GI	0.69 ± 0.27	0.64 ± 0.25	0.67 ± 0.33	0.65 ± 0.29
PI	0.67 ± 0.31	0.42 ± 0.25	0.65 ± 0.31	0.5 ± 0.31
Probing depth	1.94 ± 0.27	2.61 ± 0.44	1.57 ± 0.42	2.42 ± 0.33

**Table 3:** Sequences of qRT-PCR primers

Primers	Sequence 5’- 3’	Size: bp
GAPDH	F: CAC TGC CAA CGT GTC AGT GGT G R: GTA GCC CAG GAT GCC CTT GAG	121
IL-1β	F: GGA GCA ACA AGT GGT GTT CT R: AAA GTC CAG GCT ATA GCC GT	458
IL-6	F: CCT GAA CCT TCC AAA GAT GGC R: CTG ACC AGA AGA AGG AAT GCC	423
IL-8	F: CGA TGT CAG TGC ATA AAG ACA R: TGA ATT CTC AGC CCT CTT CAA AAA	200
IFNγ	F: CTA GGC AGC CAA CCT AAG CA R: CAG GGT CAC CTG ACA CAT TC	180
FAK	F: GAA GCA TTG GGT CGG GAA CTA R: CTC AAT GCA GTT TGG AGG TGC	146
RANKL	F: ATA CCC TGA TGA AAG GAG GA R: GGG GCT CAA TCT ATA TCT CG	202

**Fig 1:**
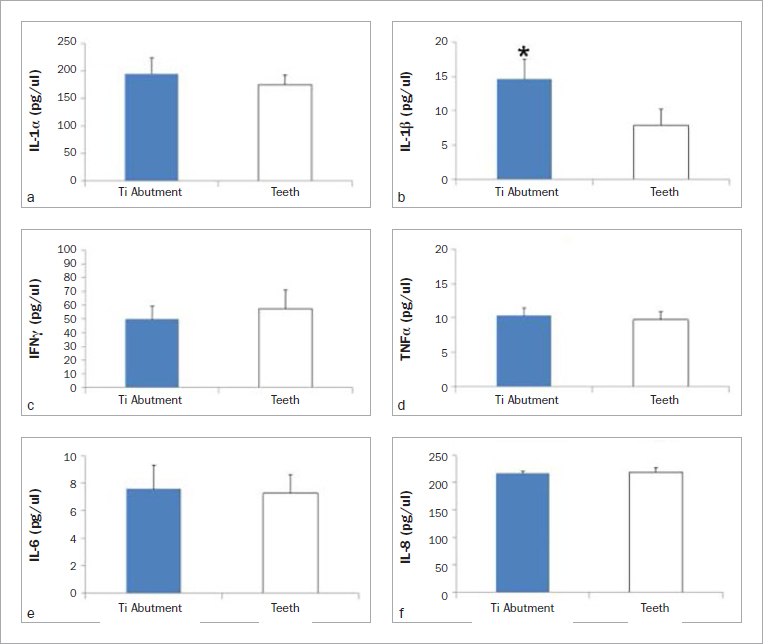
Differential cytokine expression in periodontal and peri-implant crevicular fluid at titanium abutment sites. The crevicular fluid was collected from teeth, or nine implant sites restored with titanium (Ti) abutment of the same individual. Expression of IL-1α (a), IL-1β (b), IFNγ (c), TNFα (d), IL-6 (e), and IL-8 (f) is shown. The levels of IL-1β expression in PICF are significantly higher than for natural teeth (p = 0.032) as indicated with *. ELISA was performed in triplicate and the mean represented the cytokine concentration (pg/ml) of each individual. Means and standard errors of each group are shown.

**Fig 2:**
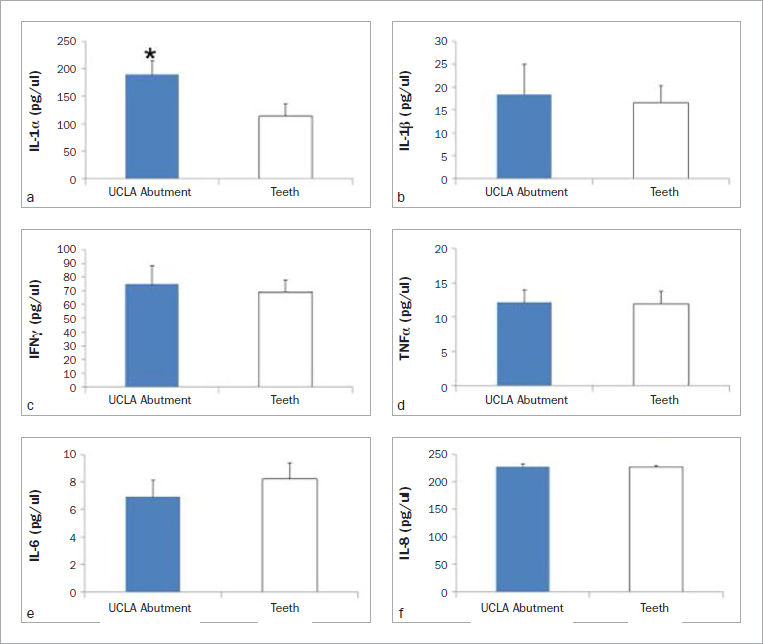
Differential cytokine expression in periodontal and peri-implant crevicular fluid at UCLA abutment sites. The crevicular fluid was collected from teeth, or ten implant sites restored with cast-gold (UCLA) abutment of the same individual. Expression of IL-1α (a), IL-1β (b), IFNγ (c), TNFα (d), IL-6 (e), and IL-8 (f) is shown. The levels of IL-1α expression in PICF are significantly higher than for natural teeth (p = 0.030) as indicated with *. ELISA was performed in triplicate and the mean represented cytokine concentration (pg/ml) of each individual. Means and standard errors of each group are shown.

**Fig 3:**
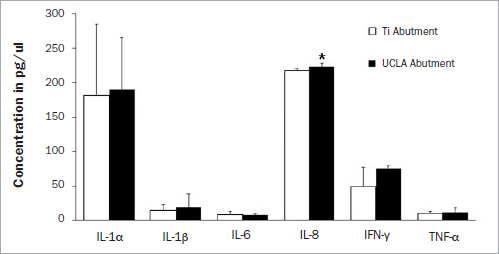
Comparison of cytokine expression in peri-implant crevicular fluid at titanium or UCLA abutment sites. The crevicular fluid was collected from nine implant sites restored with titanium (Ti), or ten implant sites restored with cast-gold (UCLA) abutment. Expression (pg/ml) of IL-1α (A), IL-1β (B), IFNγ (C), TNFα (d), IL-6 (e), and IL-8 (f) is shown. The levels of IL-8 expression in PICF from UCLA abutment are statistically significantly higher than that from titanium abutment (p = 0.003) as indicated with *. ELISA was performed in triplicate and the mean represented cytokine concentration (pg/ml) of each individual. Means and standard errors of each group are shown.

In the first part of this study, differential expression of some inflammatory cytokines was demonstrated in PICF and GCF of the same individual. The expression of inflammatory cytokines, including IL-1α, IL-1β, IL-6, IL-8, IFN-γ and TNF-α, were investigated and compared between natural teeth and dental implants. Means ± SD (pg/ml) of the inflammatory cytokines detected by ELISA in GCF or PICF from titanium (Fig 1) or UCLA abutments are shown (Fig 2). In a healthy state, most cytokines expressed in crevicular fluid were indistinguishable between the natural tooth and dental implant of the same individual, except for IL-1β and IL-1α. The paired sample t-test demonstrated a statistically significant difference in the level of IL-1β (p = 0.032) between dental implants restored with titanium abutment and natural teeth (Fig 1B); the level of IL-1α was also statistically significantly different (p = 0.030) between implants restored with UCLA abutments and natural teeth (Fig 2A). To compare the cytokine response between the implants restored with titanium or UCLA abutments, the expression of inflammatory cytokines from periodontal and peri-implant crevicular fluid was demonstrated (Fig 3). The independent t-test also showed that UCLA abutments statistically significantly increased IL-8 expression to a greater extent than that with titanium abutments (p = 0.003).

**Fig 4:**
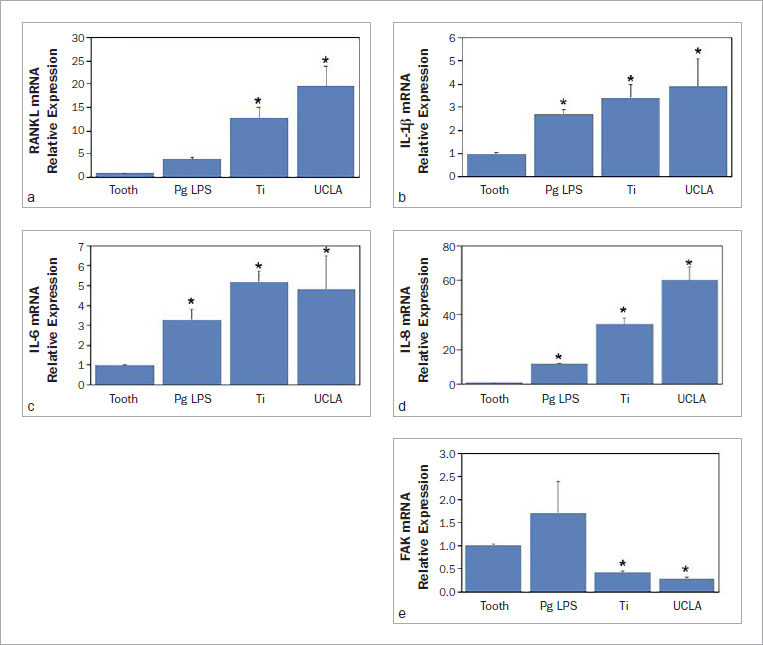
Peri-implant crevicular fluid down-regulated focal adhesion kinase mRNA in human gingival fibroblasts. HGF cultures were incubated with 0.01% volume of PICF from dental implants restored with titanium (Ti) or UCLA abutment, respectively, for 24 hours. Means of relative expression of RANKL (A), IL-1β (B), IL-6 (C), IL-8 (D), and FAK (E) detected by quantitative SYBR PCR assay from 3 independent experiments are shown. Error bars indicate standard deviation. One-way ANOVA showed statistically significant differences (p<0.05) as indicated with *.

The expression of some inflammatory cytokines differed between periodontal and peri-implant crevicular fluid. Therefore, in the second part, it was investigated whether these inflammatory cytokines affected human gingival fibroblasts. Inflammatory response to periodontal or peri-implant gingival crevicular fluid obtained from dental implants restored with titanium or UCLA abutments was examined in primary fibroblast culture (ranging from 0.005% to 0.01% volume) after 24-h incubation. The effects on mRNA levels of focal adhesion kinase (FAK), receptor activator of nuclear factor-κB ligand (RANKL), IL-1β, IL-6 and IL-8 was observed by quantitative SYBR PCR. Normal HGF culture in the growth medium was used as a negative control (data not shown). HGFs stimulated with *P. gingivalis* LPS (0.1 μg/ml) for 24 h were included for positive control. One-way ANOVA analysis (p < 0.05) revealed that PICF from both titanium and UCLA abutments statistically significantly stimulated higher expression of RANKL, IL-1β, IL-6, and IL-8 mRNA expression in human gingival fibroblasts, while FAK suppressed mRNA expression (Fig 4). The effect was dependent on concentration (data not shown).

## Discussion

Peri-implant soft-tissue sealing contributes to tissue integration and protects the vulnerable dental implants from microbial challenge. Therefore, probing around dental implants in maintenance visits or rigorous oral cleaning may compromise the tissue barrier^16^ and increase exposure to external stimuli. Normal tissue attachment appears to depend on homeostasis in host-microbe interactions, and the inflammatory mediators in gingival crevicular fluid are relevant risk indicators for disease activity.^26^ Biofilms also contribute to inflammatory reactions and recruitment of inflammatory cells. Various factors affect bacterial adhesion, such as surface free energy, hydrophilicity, surface chemistry, surface charge, roughness, and the presence of proteins.^1^ Collectively, one of the factors which contributed to the health of peri-implant tissue is fibroblast response to biofilm-induced inflammatory cytokines in gingival crevicular fluid.^31^

Gingival crevicular fluid is easily collected and contains diagnostic biomarkers. Therefore, host responses in healthy tissue can be monitored through molecules expressed in peri-implant crevicular fluid.^21^ Both IL-1α and IL-1β have been shown to be expressed in gingival crevicular fluid with inflamed periodontal tissue.^2,11^ This study demonstrated up-regulation of inflammatory cytokines, with statistically significant increases in and IL-1β, in peri-implant crevicular fluid compared to natural teeth of the same individual. Only implants placed at the crestal or subcrestal level in the anterior region were evaluated. All implants were in function from 2 to 5 years and no visible mucosal inflammation was observed in any of the patients. The differential expression of inflammatory cytokines in crevicular fluid is likely due to abutment materials.

The results of this study are consistent with previous studies that showed an elevated level of IL-1β in peri-implant crevicular fluid associated with specific oral bacteria.^21^ Apart from microorganisms, the wear of titanium surfaces may activate the inflammasome in macrophages and suppress osteogenic activity.^17^ Titanium particles and lipopolysaccharides from the oral pathogen *Porphyromonas gingivalis* stimulate the release of TNF-ɑ, IL-1β and IL-6 from THP-1 cells^10^ and potentially stimulate bone resorption.^29^ An in vitro study showed that gold-casting alloy displayed the strongest bacterial adhesion in comparison to titanium and zirconia with the same surface roughness, due to its higher polar surface energy and lower nonpolar surface energy.^14^ That study also demonstrated an increased concentration of inflammatory cytokines in yellow-gold abutments compared to titanium or zirconia abutments, but only IL-8 levels were statistically significantly different. Peri-implant mucosal defense may be strengthened and become more responsive to later pathogen challenges by recruiting higher inflammatory cell infiltration, as demonstrated by histological analysis which found gold alloy to have the highest percentage of inflammatory cellularity grades in comparison to titanium and zirconia.^23^

The interplay between microbial challenge and the inflammatory response of the host may result in an equilibrium with reversible inflammation. However, any environmental and acquired risk factors of the individual and host genetics could also activate immune dysregulation with excessive inflammation.^13^ As the surface substrates can influence cell adhesion strength, the material used for the implant abutment therefore determines both the microbial microenvironment and epithelial integration.^6^ Gingival fibroblasts around dental implants represent the first line of defense against external stimuli and play a crucial role in stimulating other inflammatory components in peri-implant tissue. It was reported that fibroblast adhesion decreased on sandblasted titanium, polished titanium, sandblasted zirconium oxide, polished zirconium oxide, gold alloy, and chrome-cobalt alloy.^22,30^ The chemical composition of gold alloy and chrome-cobalt alloys may be less compatible with gingival fibroblasts, despite similar or even higher roughnesses. The present study demonstrated that the crevicular fluid collected from dental implants stimulated RANKL, IL-8, IL-1β and TNFα mRNA in a dose-dependent manner, while FAK suppressed mRNA expression in human gingival fibroblasts when compared to the crevicular fluid collected from natural teeth. RANKL is a member of the Tumor Necrosis Factor (TNF) superfamily and is well established as the master regulator of osteoclast formation and bone resorption,^25^ while modulating inflammation and immune activation.^20^ Inflammatory responses may lead to destructive bone resorption, where RANKL up-regulation plays a crucial role in osteoclastogenesis. In addition, IL-8^28^ IL-1β^18^ and TNFα^19^ are known as the key inflammatory cytokines reported in pathologic inflammation of bone disease.^27^ Up-regulation of these inflammatory cytokines in human gingival fibroblasts suggests the potential of inflammatory mediators in peri-implant crevicular fluid to initiate the inflammatory response from human fibroblasts. This tendency appears to be higher with gold alloy than titanium abutments. IL-8 up-regulation and FAK down-regulation by gold alloy reducted fibroblast adhesion^12^ and compromised soft tissue integration. The results therefore suggested that titanium abutments are more favourable than gold alloy abutments in terms of promoting fibroblast adhesion and reducing osteoclastic activity. The increase of inflammatory cytokines may change healthy signaling to deregulated signaling in response to the surrounding microenvironment. This event may gradually result in destructive changes of connective tissue and surrounding bone.^5^

## CONCLUSIONS

Inflammatory cytokines, including IL-1α and IL-1β, detected in the crevices around healthy peri-implant tissue are more abundant than in healthy periodontal tissue. Peri-implant microenvironments have an influence on the host immune response. Peri-implant soft-tissue sealing and restorative material type, which provide a different microenvironment and oral biofilm composition, contribute to differential cytokine responses. Peri-implant crevicular fluid, especially from gold-casting alloy abutments, potentially stimulate fibroblast function to increase osteoclastic activity while decreasing fibroblast adhesion. The use of yellow-gold abutments should still be considered due to their good esthetics compared to titanium abutments, especially in the thin periodontal biotype. However, when yellow-gold abutment material is used, special attention should be paid to the patient’s oral hygiene, as the material itself can recruit inflammatory cytokines.
